# A social wellbeing approach to the gendered impacts of fisheries transition in Gujarat, India

**DOI:** 10.1007/s40152-023-00299-0

**Published:** 2023-03-31

**Authors:** Rajib Biswal, Derek Stephen Johnson

**Affiliations:** 1 Natural Resources Institute, University of Manitoba, Winnipeg, Canada; 2 Department of Anthropology, University of Manitoba, Winnipeg, Canada

**Keywords:** Social wellbeing, Gender, Change, Bag net fisheries, Gujarat

## Abstract

In this paper, we use the analytical lens of social wellbeing to interpret the history of livelihood change in the coastal village of Saiyad Rajpara in Gujarat over the past 70 years. We describe a broad narrative of transition from food scarcity to food security brought about by the introduction and intensification of bag net fishing in the village. This form of fishing has largely displaced the previous economic basis for livelihoods of uncertain daily wage labour. In a pattern common along the coast, an economy offering at best subsistence has shifted to one that is market-oriented, and which generates considerable surplus. We use the social wellbeing perspective to take stock of and order the complex effects of this transition. While the intensification of small-scale fishing in Saiyad Rajpara resulted in a general and marked material improvement in the lives of the residents of the village, the social relational benefits and subjective experience of change have been more mixed, particularly along lines of gender. A social wellbeing perspective offers an approach to fisheries governance that is more inclusive and sensitive to local experience.

## Introduction

Explicit or implicit in the small-scale fisheries literature is the spectre of change. This concern is embedded in the normative position that motivates much of the literature: small-scale fisheries make a range of societal contributions, but these benefits are threatened by long-term patterns of change that are displacing or even threating to eliminate the sector. In this paper, we accept this framing to the study of small-scale fisheries while aligning ourselves with research using a social wellbeing lens that points to how change in small-scale fisheries is made and experienced in ways that vary by social position (e.g. Coulthard 2008; Idrobo and Johnson 2020). We draw on recent arguments that emphasise the relational quality of social wellbeing (Johnson 2018; Fabinyi and Barclay 2022). From this perspective, wellbeing is seen as necessarily defined and experienced in relation to others, to place, and as varying by context. Emphasis on relationality is important not just because it underscores a central quality of human social life, but also because it links social wellbeing to similar relational qualities in other social theory approaches. Importantly, for the concern of this paper with the gendered experience of change, a relational approach to social wellbeing also provides a philosophical connection to gender theory, political ecology, and interactive governance, each of which provides complementary elements to our broader framing of change and social difference.

Our research focus concerns the fishing village of Saiyad Rajpara in Gir Somnath District. We look at men’s and women’s contrasting experiences of the major changes in their fishing economy. The social wellbeing lens allows us to highlight the mixed experience of change in Saiyad Rajpara across gender, with men’s wellbeing experience of fisheries transition less positive on the whole than women’s. The increasing importance of market relations has put tremendous pressure on the local fishers in Saiyad Rajpara through heightened competition over fishing as more boats have been chasing fewer fish. The growing commodity orientation of fisheries in Saiyad Rajpara has, however, increased the space for women’s agency as their economic opportunities have grown.

There are obvious and less obvious implications of our findings for fisheries governance in Saiyad Rajpara. Most clearly, governance that seeks to advance human wellbeing as its primary objective needs analytical tools like the social wellbeing perspective that can tease out the complex and varied experience of change. Fisheries governance in Gujarat has always been production oriented, with insufficient consideration for the distributional aspects of development (Johnson 2001). Our gender analysis shows how fisheries governance, admittedly bluntly applied in this case, may have complex and sometimes unexpected outcomes across different social groups. The more subtle implication of this observation is to find ways to make governance more relational, or transdisciplinary, in the sense of building better institutional capacity for knowledge sharing and bridging differences amongst participants in fisheries, including formal fisheries governors.

## Wellbeing, change, and gender in small-scale fisheries

Change is an inevitable condition of human existence and, as such, has been a central if not always explicit element in social science from its earliest days. Often, it is the most intense kinds of change that garner attention. In social-ecological systems terms, these are the shocks of revolution, flood, untimely death, and so on that repeatedly punctuate life. Change also moves in slower and sometimes more benign ways, of course, and the regularities of experience like seasonality and short-, medium-, and long-term trends such as inflation, shifts in norms, or climate change are as important in shaping the fabric of human life.

In very general terms, and over an extended time scale, small-scale fisheries globally have experienced a trend towards technological intensification and an increasing commodity orientation. This transition has been driven by growing demand for seafood products globally and locally, and by state policies aimed to facilitate economic growth to service expanding markets (del Corral 2008; Stevens et al. 2014; Pitcher and Lam 2015; FAO 2020; Armitage and Johnson 2006). The common story for small-scale fisheries that experience this trend is heightened competition with industrial fishing and, increasingly, other users of coastal and ocean space. In recent years, the term blue economy has come to represent the strategy for coastal and maritime development that is driving the increasing congestion of maritime space and leading to further deterioration of resource availability and access for small-scale users (Barbesgaard 2018; Bennett et al. 2019). Much like globalisation, the blue economy description of change is useful analytically to frame large-scale processes of development and change. To capture the particularities of experience of small-scale fishers in different regions and places globally, additional analytical tools are needed. As theory and method, social wellbeing provides one approach to describe and interpret the variability of small-scale fisher experience of change in places like Saiyad Rajpara.

The social wellbeing framework has become an important reference approach to understand complex attachments to, and societal contributions of, fishing (e.g. Britton and Coulthard 2013; Weeratunge et al. 2014; Voyer et al. 2015; Galappaththi et al. 2021). Our paper builds on the observation in this literature that the societal value of fisheries is a relational phenomenon; what wellbeing means, and how fisheries contribute to wellbeing, have to be understood as emerging in the context of particular historical transitions and social, economic, and political relations (Coulthard 2012; ref suppressed). Framed in this way, social wellbeing is an analytical bridge between larger scale phenomena, change, and lived human experience. The core commitment to a relational framing of life links social wellbeing to similar philosophical orientations in the range of other social science approaches that we mention above.

The social wellbeing perspective provides a philosophically grounded approach to meaning and thriving in human life (Gough and McGregor 2007) that has been translated into practical methods for understanding diverse experiences of change (Coulthard et al. 2015). Social wellbeing conceptualises wellbeing in multi-dimensional terms. This orientation derives from social wellbeing’s emergence from debates around poverty in international development studies and builds particularly on Amartya Sen’s critiques of conceptualisations of poverty that reduce it to economic want (Gough et al. 2007). Sen argues instead that poverty should be understood as a failure of capabilities and thus an inability to pursue a life that one sees as meaningful (Sen 1999; Coulthard 2012). Building on Sen, the social wellbeing approach sees poverty, or illbeing, as a failure of material, relational, and subjective capabilities. These three dimensions of human flourishing can thus usefully frame inquiries into the degree to which individuals and human groups are doing well, and how change is affecting wellbeing (Breslow et al. 2016).

The material aspect of the social wellbeing framework identifies major contributions in areas such as food security, employment and income, or standard of living. The relational dimension explores the relationships and social networks which connect individuals with the wider social context. It includes areas such as gender relations, political and governance relations, and any other relationship fishers might have that enhances or constrains their ability to pursue their wellbeing. As White (2009) emphasises, social relationships not only shape an individual’s wellbeing in the capacity sense of social capital, but also point to the relational nature of wellbeing. Perceptions of wellbeing, whether objective or subjective, vary depending on with whom and in what context one is engaged (White 2009). Subjective wellbeing is a person’s assessment how they perceive or value their life (Camfield 2006). While subjective wellbeing is intangible (Smith and Clay 2010), the material and relational dimensions of a social wellbeing approach are more readily observed, although they do not always perfectly match individual subjective perceptions of wellbeing or illbeing (Biswas-Diener and Diener 2001). As concerns fisheries, the subjective and relational dimensions of social wellbeing point to how fishing needs to be understood beyond conventional cost–benefit terms and rather should be considered, as many maritime anthropologists describe, as ‘a way of life’ for the members of coastal communities (Pollnac and Poggie 2008; Smith and Clay 2010).

Building on the work of Trimble and Johnson (2013) and others (Coulthard 2012; Lalancette 2017; Fabinyi and Barclay 2022), we see social wellbeing as a valuable tool that can complement political ecology, gender theory, interactive governance, social ecological systems, collective action, and other approaches to understand experiences of and responses to change. Change originates from and has effects across different scales. It shapes and constrains action, but also offers variable opportunities for agency within prevailing social and economic structures. The material, relational, and subjective reference points of social wellbeing provide a means to assess how change threatens or advantages differently positioned individuals and groups.

Work in fisheries is gendered in varying ways, with women normally playing a greater role in land-based activities and men in harvesting. Men typically dominate larger-scale and more commercialised activities with women commonly involved in processing labour, small-scale trading, and petty retail (Weeratunge et al. 2010; Hapke and Ayyankeril 2004). Contemporary research on gender in fisheries points to the intersectional complexities of gender relations where the performance of gender is shaped by class, ethnicity, caste, and other social positions (Galappaththi et al. 2021).

It has long been recognised that gender is a primary basis for the socio-economic organisation of work in fisheries and a fundamental filter for the effects of change (MacDonald and Connelley 1990; Ram 1992). The commercialisation of fisheries through the development of new markets and export-oriented production has created many employment opportunities for men and women in fishing communities (Kurien 2004; Béné 2006) while also influencing the role and position of men and women within the fish supply chain (Thorpe and Bennett 2004; Neis et al. 2005; Ram-Bidesi 2008; Tindall and Holvoet 2008; Dunaway 2013). Transitions have implications for the gender division of labour in fisheries. Men’s and women’s economic activities have diversified (Acheson 1981; Aldon et al. 2011) and globalisation has influenced the space in which men and women engage in fishing activities (Miller 1993; Bennett 2005; Hapke 2012). The gendered effects of change are also situated and intersectional (Frangoudes et al. 2019). In simple terms, change is led and experienced by women and men in ways that vary according to class, ethnicity, place, and experience, amongst other factors. Holly Hapke’s research in Kerala on how women’s possibilities in fish trading vary by religious identity, age, economic status, and location clearly illustrates this point (Hapke and Ayyankeril 2004, 2018).

The research on Saiyad Rajpara described in this paper builds on studies that have tried to trace economic, social, and ecological change and governance in the marine fisheries of Gujarat State, India (Johnson 2002; Johnson 2009; Biswal et al. 2017; Nair and Baxi 2022). These studies take a political ecology perspective in which change is understood as the product of multi-faceted, contextually specific, and contested relations amongst different actors and groups, including non-humans. This work sees change relationally in terms of the importance of specific conditions and variable human experiences.

Broader research on Gujarat highlights the state’s unique fisheries development trajectory. State marine fisheries support a vibrant and diverse fishing population but state fisheries development policy from the 1960s was premised on mechanisation and export promotion without effective institution building. From the 1990s onwards, there have been clear indications of overfishing, with resultant instability in fishers’ livelihoods. Small-scale fishers have voiced considerable dissatisfaction at undue pressure from large-scale mechanised trawler fishers in the state.

The experience of the inhabitants of Saiyad Rajpara mirrors this story of resource crisis, and governance is an important consideration given how governance choices and governance arrangements for the Gujarat coast have been a crucial driver of material and subjective changes in the fishery (Johnson et al. 2018). We conceive of governance in the terms of interactive governance theory, which, we argue has a similar relational orientation as the social wellbeing approach in how it sees governance as emerging within particular contexts and participation varying by social position (Jentoft and Chuenpagdee 2009). Interactive governance holds that governance is the contested and dynamic process through which different groups, state and non-state, interact across multiple scales to more or less effectively manage access to and use of resources (Kooiman and Bavinck 2005). From this perspective, Saiyad Rajpara can be understood as being subject to legal plural or mixed-regime governance where multiple state and non-state actors play various roles in the management of the local bag net fishery. These actors often poorly coordinate their efforts (Berkes and Fast 2005; Bavinck et al. 2012; Biswal et al. 2017). We address the implications of this form of governance particularly through the relational dimension of the social wellbeing approach while recognising the effects that quality of governance has on material and subjective dimensions of wellbeing as well. We stress  how the effects of fisheries development and governance in Saiyad Rajpara must be understood in gendered terms that are sensitive to position, power, and social status (Hapke and Ayyankeril 2004; Power 2005).

## Context and methods

Gir Somnath District was created in 2013 out of the subdivision of Junagadh District. Gir Somnath has the highest marine fish production of any district in Gujarat. In 2020, it contributed a total of 261,000 tonnes of the Gujarat’s total catch of 532,000 tonnes (Central Marine Fisheries Research Institute (CMFRI) Annual Report 2020). Veraval is the district headquarters and is a major hub for fish processing and exports for the entire western Indian region. Favourable biophysical conditions such as the largest continental shelf of all Indian states and the productive gulfs of Kachchh and Khambat have benefitted the state’s fisheries.

**Fig. 1:**
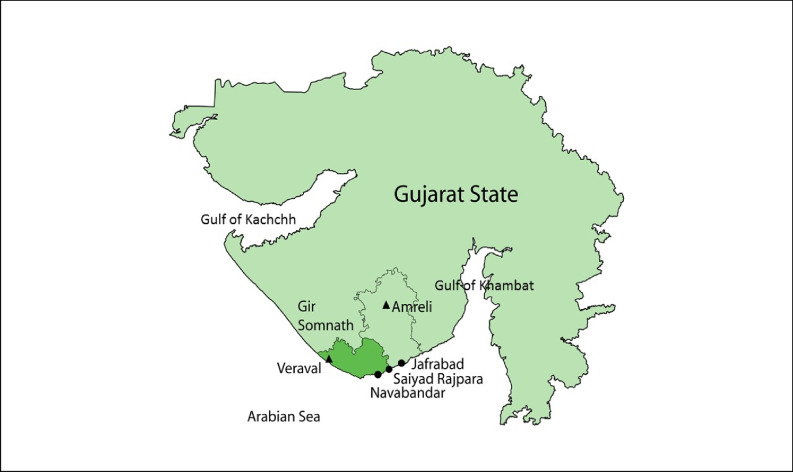
Gir Somnath district within the state of Gujarat

The fishery of Gir Somnath can be categorised into dried-fish and fresh-fish zones. Fishing practices and market systems are the main features that differentiate the two fisheries within the district. The dried-fish zone, on the eastern coast of Gir Somnath from Navabandar to Jafrabad (Fig. 1), is focused on fish caught using bag nets and then dried. The funnel-shaped bag net is fixed gear, with a wide mouth and closed cod-end with small mesh size that is well-adapted to the relatively shallow seabed with high water currents of eastern Gir Somnath district. The bag net fishery is arguably semi-mechanised, or intermediary between the two standard categories of fishing intensity in India of non-mechanised and mechanised (Jadav 2018), and this stationary fishing gear is comparatively more cost effective than other active gears such as trawl nets or seine nets (Biswal 2015). According to the CMFRI annual report (2020), bag netters (mechanised and outboard) are the second largest contributors (26.71%) to annual landings in Gujarat after trawl netters. Traditionally, Bombay duck (*Harpodon nehereus*) and ribbon fish (*Lepturacanthus savala*) were the two main species harvested, processed, and exported to other places within the district as well as to other places including neighbouring states, and some export markets. Paste shrimps (*Acetes indicus*) and other ‘trash’ fish (mostly juveniles which come as bycatch) are relatively new additions to the dried-fish economy due to the access to new markets, as are larger fresh fish species such as sharks, rays, and catfish.

Saiyad Rajpara has a fine natural harbour. It is a comparatively homogeneous village, as more than 90% of its inhabitants belong to the relatively low status Koli caste. A majority of the Kolis are engaged in fishing with a few households in farming and very few engaged in either small business or employed elsewhere. Dalits are the second largest community in this village. Dalits are mostly daily wage earners and part of the fish supply chain. There are a few Muslim households, mainly involved in either the dried-fish trade or petty business. The village economy predominantly depends on the bag net fishery.

This paper is based on an ethnographic study which was conducted over 4 months during the last quarter of 2014. The first author has maintained his connection to Saiyad Rajpara since the he conducted the fieldwork, but the main arguments in this paper are grounded in the experience of members of the village in the years up to 2014. The research was largely undertaken in Saiyad Rajpara village by the first author with occasional visits to the neighbouring harbours, the sub-regional office of the Fisheries Department at Jafrabad, and to the regional fisheries office and district headquarters at Veraval. Living within the Koli fishing community and participating in their daily activities provided an important ethnographic appreciation for fishers’ daily life and experience in Saiyad Rajpara (Davis and Henze 1998; Reeves et al. 2013**)**.

The paper’s qualitative data were generated primarily through empirical research and secondary sources. Empirical data were collected through triangulation which included multiple sources of data generation (Hanson et al. 2011; Petty et al. 2012). A multi-dimensional social wellbeing approach was used as the main framework and data were generated through semi-structured interviews, participant observation, informal discussions, and field notes. The Relational Wellbeing Assessment tool was used in assessing the important relationships fishers share with fellow fishers and crew members, traders, middlemen, or the state institution (Coulthard et al. 2015). This tool asks research participants to identify the most important relationships in their lives and to rank their satisfaction with them.

A total of 69 respondents, including 38 fishers, boat owners, saleswomen, women wage earners, middlemen, fish traders, important people from the community, and government employees, were selected and interviewed through purposive sampling. A major source of empirical data on food consumption was collected through participant observation, informal discussions, and through active participation in various cultural activities and celebrations. The first author also joined two fishing trips, which were useful not only to experience fishing activities but also to understand more directly the risk and stress of fishing. The qualitative data, generated through multiple sources, were then transcribed, translated, and organised. NVIVO software was used in the analysis of data and identification of various emerging themes. Field notes and literature collected from the secondary sources helped to fill the gaps in data.

This research was designed as a study of changing practices of fishing and governance in the bag net fishery of Saiyad Rajpara and did not include an explicit gender orientation. In hindsight, this was an important oversight and meant that men’s and women’s perspectives were poorly balanced in the study. Formal interviews of women were limited to seven. We also conducted two focus group discussions with 10 women and the first author interacted with women through the ethnographic component of the research. These interactions were necessarily restricted by local factors, however. Fishing-related space in Saiyad Rajpara has a nearly ubiquitous male presence and prevailing norms place limits on the access of male researchers to free and quality interaction with women. In the rural Gujarat context, it is not common for women to talk to a male outsider. Despite these limitations in study design and access, however, we did gain significant and novel insights from our research and data about the gendered experience of change.

## Transition in the development of Saiyad Rajpara’s fishery

### The initial phase of transition

Older residents of Saiyad Rajpara report that life for Koli residents was difficult and uncertain approximately 70 years before research was conducted. Up to that time, many people suffered due to chronic hunger and malnutrition. Fishing was not the main livelihood activity because the Kolis were not traditional fishers in that region. After India’s independence in 1947 and the departure of Muslim rulers, Kolis were left on their own with limited sources of income. Given the saline environment and limited and unirrigated cultivable land, agriculture was not a particularly viable option for the locals. Many Kolis were engaged in daily wage labour such as stone breaking or clearing fields of fodder in nearby areas. Saiyad Rajpara residents struggled to get two square meals per day after a day’s hard work. One of the inhabitants during an informal conversation stated that only a few people would eat fish at that time due to the dominant vegetarian culture^1^ and fish were not easily available in the local market. Most people would just eat seasonal vegetables and pulses, when they could afford them, with flatbread made of millet flour.

The socio-economic life of the Kolis in Saiyad Rajpara underwent a major upheaval after the arrival of the ‘Machhi’ people from the southern part of Gujarat state about 70 years ago. The ‘Machhis’ are a traditional South Gujarat fishing caste, and they introduced the bag net fishery into the region. The Machhis found the natural harbour of Saiyad Rajpara and the relatively shallow region of the Arabian Sea suitable for bag net fishing. Initially, the Machhis hired Kolis as daily wage labourers to perform a range of tasks such as loading and unloading supplies and catch at the harbour. Gradually, the Kolis started working as crew members when there was a crew shortage. Over the years, the Kolis acquired the skill of fishing from the Machhis. Fishing by the Machhis ceased locally approximately around 2005, after a period of decline, as the younger Machhi generation was no longer interested in bag net fishing and many of the young Machhis feel they can earn better incomes by working in the more productive trawl sector. Over the period when Machhis were in Saiyad Rajpara, the Kolis gradually adopted bag net fishing as their major livelihood and have fully taken it over since the Machhis withdrew in the 1990s and 2000s.

A bag net (or *dol* net in Gujarati) is a large thick polypropylene fibre net about 25 metres long, which is attached by heavy ropes to two large metal pipes which are sunk deep into the sea bottom, serving as anchors. Bag net fishing is practiced in shallow areas where the water current is high. The broad mouth of the bag net with large mesh size net is placed facing against the current. The net narrows and the mesh size shrinks towards the tail in order to capture a variety of sizes of fish. Unlike trawl nets, bag nets are a stationary gear. Bag nets target Bombay duck, prawns, ribbon fish, and many other species.

In the early years of bag net fishing, Saiyad Rajpara Kolis did not have instruments such as high-frequency radios and GPS and followed the indigenous methods of fishing practiced by the Machhis. The time of fishing would depend on the wind and the season did not start until October, in contrast to the present where it now starts in late August. While men fished, women were involved in processing, drying, and selling of fish. After 2 days at sea, fishers would typically return with a hold full of fish. Despite the availability of a variety of species, fishers focussed primarily on Bombay duck, golden anchovy (Colia dussumieri), and ribbon fish. There was no demand for other species in the local market. Fishers’ pace of work was relatively easy and low risk, with the availability of plenty of fish and quite short trips. On the other hand, the life of women was tough as the processing, drying, and fermentation of these fish was time-consuming and required a great deal of hard physical labour. Without the availability of cold storage facilities, women’s work was crucial to ensuring that fish were dried before they could spoil. Once fish were dried completely, women were responsible for selling them. As there was no transportation available, women would walk miles in the sun with a basket on their head to sell their dried fish in neighbouring villages. There was no fixed time for women to process fish and they would often work at night depending on the arrival of fishing boats. With the advent of fishing as a livelihood focus, Koli fishers and their families began to make a decent living and food scarcity was no longer an issue. In the meantime, fish became central to the diet of Koli households in Saiyad Rajpara.

### The boom phase

If the gradual transition to Koli control of the bag net fishery from the 1970s to the 1990s was accompanied by greater economic and nutritional security, in the early 2000s, a completely new sort of economic possibility became apparent with the advent of the fresh fish trade. This was led initially by a private company from the nearby town of Una called Visti, which had commenced trading dried fish in Saiyad Rajpara in the early 1990s. After a period of building rapport in Saiyad Rajpara, Visti began to offer boat owners monetary advances at the start of the monsoon in exchange for the right to buy their fresh fish catches. Visti had a monopoly on the fresh fish market for several years, after which other traders began to join the fresh fish business. By 2014, the arrival of many other fish traders in Saiyad Rajpara had created more competition as well as opportunities for the locals. Currently, there are 15 fish traders operating in Saiyad Rajpara (Personal communication, June 07, 2018).

The arrival of Visti and the other fresh fish traders allowed for a diversification of market opportunities for fishers, growth in demand that exceeded existing fishing capacity, increased prices for fish, and more stable and locally available credit. All of this triggered much greater profitability of the fishery. The result was a boom that lasted from Visti’s arrival until around 2010–2011.

During this initial boom phase, boat numbers in Saiyad Rajpara skyrocketed from 100 to approximately 450 and the total number of fishers (primarily engaged in harvesting) from less than 1000 to approximately 3000. The access to new market opportunities and income also contributed to the modernisation of the fishing fleet (see the ‘Material wellbeing’ section for more detail). Furthermore, the access to new markets shifted the fishery to high value pelagic fish, rendering the traditional harvesting of Bombay duck, ribbon fish, and golden anchovy secondary in importance (Johnson and Sathyapalan 2006; Biswal, 2015; Johnson et al. 2018). The newly in demand fresh fish included species such as Jew fish (*Protonibea diacanthus*), pomfret (*Pampus argenteus* and *Formio niger*), shrimp, prawn, lobster, mackerel (*Rastrelliger kanagurtra*), and shark. The new fresh fish economy also provided new storage and transportation possibilities. The access to new markets has similar effects for women as described by Rubinoff 1999, Hapke 2001, and Ram-Bidesi (2008). It created new employment opportunities for local women, which not only provided them greater financial liberty but also enhanced their mobility.

### The post-boom phase

In the years following 2010–2011, the exuberance of the boom evaporated as a number of significant economic, social, and ecological challenges emerged. As has occurred elsewhere (del Corral 2008; Standal and Hersoug 2015), fishers reported that the bag net fishery of Saiyad Rajpara is experiencing an intensification of effort in terms of increasing number of fishing trips, and an escalation in the average number of fishing days per year. These changes are driven by the increasing scarcity of fish and competition between the much greater numbers of boats. The bag net fishery has expanded to more distant and deeper waters, which has put Saiyad Rajpara’s fishers into increasing spatial conflict with other fishers from Gujarat and Maharashtra. Loss of gear and the threat of collision at sea are an increasing problem. These governance problems in the fishery have been exacerbated by a crisis in the village’s Koli caste management body, the *samaj*. In 2012, the refusal of some members of the village to follow the commands of the *samaj* leader, the *patel*, led to the collapse of that institution. It has not met since. The absence of the *samaj* in local affairs has meant that Saiyad Rajpara fishers no longer have the organisational capacity to effectively represent themselves as a corporate group to the Gujarat Department of Fisheries or to other government bodies that might be able to help them address governance needs such as conflict with vessels from other ports. The fishers also now lack a body that can act as a counterweight against the outside merchants who are central to the economic life of fishers in the village.

Various authors have pointed out the connection between access to the market and the depletion of natural resources (Cinner and McClanahan 2006; Brewer et al. 2013; Robinson et al. 2020). These conditions, in turn, may have a negative impact on producer communities (Reed et al. 2013; Robinson et al. 2020). As documented by several researchers (e.g. Johnson 2011; Doyon 2015; Robinson et al. 2020), increase in the market value for fish can adversely affect the health of marine resources. Consistent with this evidence and these arguments, the respondents we interviewed unanimously mentioned that the quantity of catch, not only for the high-valued fish such as jew fish, lobster, and pomfret but also overall fish catch has drastically declined in recent years. Fishers in Saiyad Rajpara have come increasingly to depend on catching paste shrimps (*Acetes indicus*) and other low-value fish, which are mainly used for fish meal, to recover their operational costs.

The transition of the Saiyad Rajpara bag net fishery from a subsistence-based to a profit-oriented market-driven orientation has had a range of important impacts on the Koli population of the village. We structure our analysis of the varying and contradictory effects of that shift through reference to the three-dimensional social wellbeing perspective.

## The effects of transition on wellbeing in Saiyad Rajpara’s bag net fishery

### Material wellbeing

#### Fishery modernisation

The transition of the bag net fishery as a result of access to new markets has created ample opportunities for the fishing industry itself. The access to new markets and collaboration with fish traders have resulted in the availability of greater credit and investment in the local fishery. Almost 95% (*n* = 38) of fishers admitted that the access to advance credit through the market has helped the fishers survive during the slack monsoon period.

The new investment in the fishery helped to modernise fishing. Motorised boats have expanded fishers’ horizons beyond traditional boundaries; fishers do not have to rely on the wind or a particular season to start fishing. Local fishers now have access to modern technologies such as winches instead of having to manually haul in nets. GPS is a great help to identify directions and the position of fishing boats. Fishers no longer depend on the compass or the sun to navigate while fishing. High-frequency radios have improved communications and coordination, important improvements in this risky profession. Fishers can get weather-related information through these radios. If there is a technical problem with the engine or an accident, fishers can call for help. Fishers use LED lights which have increased their efficiency to fish at night and also to avoid accidents due to poor visibility. In a nutshell, the use of new technologies has increased the efficiency and safety of Saiyad Rajapara’s bag net fishery, similar to what Santha (2008) observed in Kerala.

#### Dietary habits

**Fig. 2:**
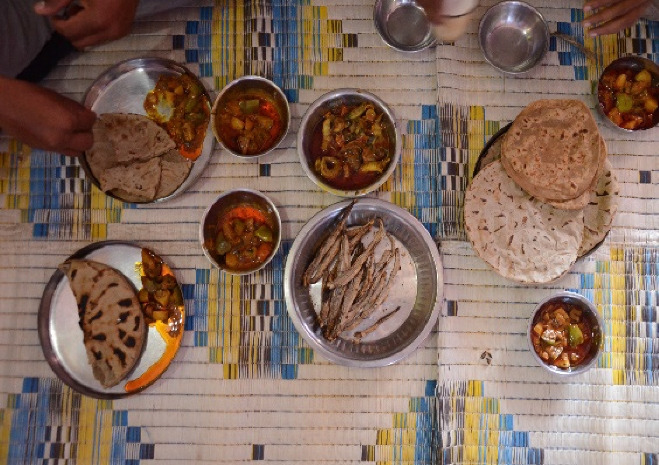
Fish as a major dietary item for the Koli community. Photo Credit: Derek Johnson

The inception of the bag net fishery influenced the food habits of the residents of Saiyad Rajpara. The once largely vegetarian community has now been transformed into a predominately fish-eating community and fish dishes now dominate everyday diets. Figure 2 shows a variety of fish items such as roasted Bombay duck, prawn curry, traditional millet chapattis, and some vegetable curry, served during lunch by a Koli family.

In contrast to places (Scanlan 2003; Deb et al. 2015) where women or young girls are discriminated against in terms of access to nutritious food such as fish, a high proportion of Koli research participants in Saiyad Rajpara stated that the distribution of food is equitable. All but one respondent out of sample of 38 stated that women in their households have equal access to nutritious and high-value fish. Women often get fish either from their places of work or receive it from family and friends while the men are away.

#### Food security

As the bag net fishery in Saiyad Rajpara has grown over the years, food security has become much more assured. Villagers now have access to surplus fish for consumption. In an echo of the findings of Kawarazuka and Béné (2011), all the fishers interviewed (*n* = 38) in Saiyad Rajpara mentioned that they enjoy eating fresh fish and that their occupation provides an opportunity to eat different kinds of fresh fish, which otherwise would be difficult for them to buy from the local market. Informant SR 17 shared, ‘We all take some fresh harvest home for our own consumption when we return from the harbour. Further, from the money we earn from selling our catch, we buy groceries, and other food items throughout the year. I think I make a decent living out of fishing’. Fishers in the village also noted that they share surplus fish with their friends and relatives when they get a good catch in order to maintain social bonds. This confirms the common observation that food security is not just about access to food but also includes the fulfilment of culturally accepted food preferences and social obligations (Belton and Thilsted 2014).

In addition to direct subsistence use, fish also contribute to food security indirectly (Kent 1997; Ram-Bidesi 2008; Jentoft and Eide 2011; Kawarazuka and Béné 2011). This observation applies also in Saiyad Rajpara, where many families have surplus fish and they sell those to the retail saleswomen in the village to get some cash to buy other food items such as food grains, cooking oil, and spices besides other daily requirements. Fish flow into household from men whose work as crew earns them a share of the catch or from women and children, who get fish from a variety of sources. Households sell surplus fish beyond what they can consume or refrigerate (for those with refrigerators) in the local fish market. We observed the generosity of the community during our fieldwork where many households shared their surplus fish with their friends, relatives, neighbours, and with some poorer households. We also witnessed the contribution of cooked and roasted fish made by some of the women from neighbouring households when a woman in a poor household delivered a baby while her husband was away at sea. This validates our finding that availability of, access to, and stability of food have improved markedly since the 1970s.

#### Employment

**Fig. 3:**
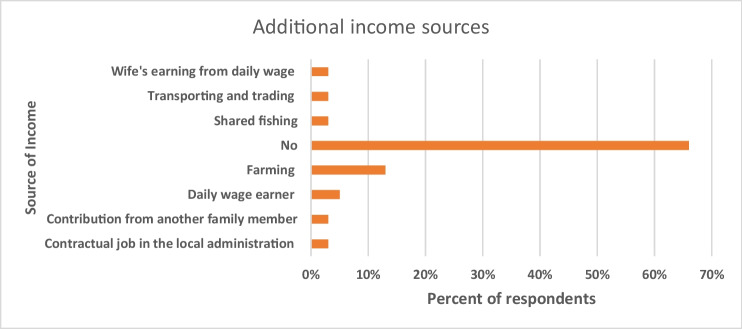
Fishers’ additional income source in Saiyad Rajpara

The modernisation of the Saiyad Rajpara fishery and its integration into new markets led to employment creation in the village. Like elsewhere (Vogt et al. 2010), the expansion of market opportunities and readily available financing for investment in Saiyad Rajpara have turned the local fishery into a thriving industry. This led to a significant increase in the number of boats from 100 to 450 in the decade to 2015. With the sharp increase in the number of boats, the bag net fishery has created more employment opportunities for local people, but also economic specialisation. Out of 38 fishers interviewed, 66% of fishers mentioned that they do not have any other source of income than fishing or fishing-related activities (Fig. 3).

Fishing is about more than just harvesting fish. It entails pre-harvest preparation and post-harvest activities such as cleaning and processing, drying, and finally selling. While the catching of fish is primarily a male-dominated activity in Saiyad Rajpara, many women are hired by traders to perform post-harvest tasks such as sorting and cleaning of fish. This is a common gender division of labour in fisheries (Bennett 2005; Ngwenya and Mosepele 2007). During discussion, the women’s group shared, ‘In earlier times, there was limited opportunity for women. Women would have to work hard for a bare minimum wage. Things have changed at present. Our daughters are gainfully employed in the fish processing units and earning much more than us’. This complementary gender division of labour provides a way to raise overall income (Hapke 2001; Hapke and Ayyankeril 2004; Bird 2007). Some men are employed in physically demanding work such as loading and unloading of fish and supplies, carrying ice, and putting it into crushing machines. Most of the fish processors and traders hire people on a yearly contract, which provides a secure income to many households in the village. Most of the women who work in the fishery are the family members of local fishers and their income is an addition to the family income.

#### Standard of living

The bag net fishery in Saiyad Rajpara has transformed the local economy from providing only a poor wage for participants to the basis for a prospering fishing village. Many fishers can afford a relatively better standard of living in comparison to that which prevailed as recently as the 1990s (Johnson 2001). During the first author’s stay in the community, he observed the standard of living of the inhabitants in Saiyad Rajpara, which seemed to be visibly better than some other nearby communities where the number of concrete-made houses was relatively low. During informal interactions, an old fisherman shared that more than eighty percent of inhabitants now live in concrete houses that were previously not affordable to them. According to the women’s group, ‘Currently, we have access to electricity, tap water, and there is an electric grinding machine to make flour. Earlier we had mud houses but now we live in concrete houses. At least we do not have to plaster the mud walls on a regular basis anymore, which saves time and eases our workload’. Most households had motorbikes and some households had transport autorickshaws when the study was conducted in 2014. Cell phones have become a crucial and common communication device for the younger generation and are also helpful for the boat owners to contact the crew prior to the departure of their fishing boats. There is a significant increase in the number of local businesses at the harbour, for example, from two or three tea stalls to approximately 15 businesses including a restaurant, many tea and snack shops, an auto parts store, and a few beverage shops at the harbour. In addition, some of the fishers have become sufficiently well off to be able to afford better education for their children by sending them to schools and colleges elsewhere. The significant contribution of the bag net fishery to local economy can be equated to the shift to cash cropping in agriculture (Béné et al. 2009). The gradual transition from an agriculturally based economy to traditional fishing and then finally to a market-oriented fishery has contributed significantly to the material quality of life of the Saiyad Rajpara inhabitants.

### Relational wellbeing

**Fig. 4:**
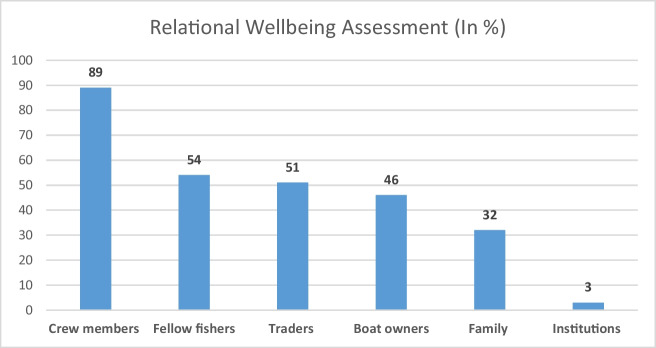
Fishers’ assessment of most important relationships

While access to new markets has created many economic opportunities for the local fishery in Saiyad Rajpara, the social relational consequences of change have been mixed. Consistent with observations in other areas of the world (Stevens et al. 2014; Pitcher and Lam 2015), fisheries modernisation has led to a prioritisation of economic relations. As Fig. 4 illustrates, our relational wellbeing assessment shows how fishers (*n* = 38) in Saiyad Rajpara have come to prioritise relationships appropriate to their occupational needs. The six columns in the figure represent different categories of actors that are involved in Saiyad Rajpara’s fishery. The percentage associated with each column represents the proportion of respondents who ranked  that actor category as one the three most important relationships in their lives. The relationship with fish traders, who are the fishery’s market intermediaries, is the third most highly ranked (51%) category. Relations with fish traders are thus seen as more important than those with family (32%) and local institutions (3%). Local institutions in this context include primarily government institutions such as the Fisheries Department, local administration, and any subsidiary of government-owned financial institution. Despite getting financial support from fish traders, the relationship between fishers and fish traders is not ideal.

During the first author’s four month long fieldwork, he became aware of the frustration of fishers due to their perceived exploitation by fish traders. Many fishers, particularly those who are in debt, do not have a voice in the decision-making process. Fishers who receive an advance from fish traders are subject to intense pressure to catch high-value fish to fulfil market demand. Informant SR20 shared, ‘I take credit from one of the fish traders when I have a loss. I can get a credit up to CAD$10,000 for eight months and in return I must supply all our fresh harvest to them at the lower price quoted by the trader’. Similarly, informants SR06 and SR14 shared, ‘If we have credit from traders, we make additional effort to harvest fresh fish and once we come back to the harbour, we have to give all our fresh harvest to the creditors’. Fishers, therefore, are not very happy with the overall situation, but they have come to rely on this financial arrangement with fish traders to continue fishing.

The paucity of effective engagement by the state, including the Fisheries Department, is indicated by the failure of fishers to recognise relations with state agencies as contributing to their wellbeing (Fig. 4). Strong relations with crew are also clearly of major importance. Fishing trips have become more frequent and relatively longer in the post-boom period and risk at sea has consequently increased. As fishers face many challenges while fishing and feel a lack of any external support by outside agencies, strong male bonding has been a coping mechanism. Crew members get substantial psychological support from each other. We observed that during social events such as festivals or weddings, many young crew members prefer to spend time with each other rather than spending time with family members. Figure 4 also shows the relationship of fishers with their families as the second last in the relational wellbeing framework results. Family is important to fishers to meet their social needs, but its relative importance is lower in the occupational context. Gender dynamics amongst fishers are an important dimension of work in the fishery, as explained in the next section.

The increasing importance of the external fish trade has weakened the relationship between the local institutions and fishers in Saiyad Rajpara. There was no functioning local institution (the s*amaj*) nor a leader (the *patel*) to manage the harbour when the research was conducted in 2014. Fishers said that they were no longer interested in the revival of these traditional governance institutions. In the absence of the *samaj* and a *patel* from the fishing community, fishers feel emancipated, but they also are more vulnerable to longstanding governance issues such as the intrusion of outsiders into their marine territory or exploitation by fish traders. These issues have considerable impact on fishers, but there is no longer a functioning local institutional mechanism to address them, or to coordinate with the state agencies to safeguard fishers’ rights or to advocate with fish traders on behalf of fishers.

### Reinforcing relationships and relational tensions

While the intensification of fishing and the longer duration of fishing trips have increased fishers’ risk, it has also reinforced their relationships with fellow fishers and with others in the community. Informant SR 26 shared, ‘In ordert to start a business in Saiyad Rajpara, a person must have a good relationship with an influential person. Second, to have access to credit, one must have good relations with the local traders. Finally, one must have a very good relationship with fellow fishers and crew members in case of emergencies and so that everyone will work hard together for a good harvest’. The exceptionally risky work environment and mutual dependency on fellow crew members while fishing contribute to solidarity amongst fishers (Pretty and Ward 2001). The bonding amongst fishers goes beyond work (Ommer 1999) as 48% (*n* = 38) of fishers interviewed mentioned that they spend most of their free time with fellow fishermen instead of staying at home or spending time with their family members. Despite spending much of their free time with fellow fishers, however, family is the first contact point for many fishers for their social as well as occupational needs. In case of a work-related or financial emergencies, many fishers prefer to rely on their family members, and family provides land-based social and emotional support to fishers. Fishers’ relationships vary depending on their age. Many young fishers (younger than 30) prefer to spend time with their fellow crew members or fellow fishers even when ashore. Unlike the young fishers, fishers who are married and have children tend to prioritise spending time with their family members.

Some fishers also indulge in alcohol and gambling as stress-relieving activities due to long working hours and insufficient rest. While sharing his frustration, informant SR05, an unmarried fisherman, shared, ‘I drink a lot to unwind myself. That’s why I am struggling to find a bride in this village’. Overwork and exhaustion have increasingly become an issue during the post-boom phase. Addictions and gambling have strained relationships between men and women within many fishing families of Saiyad Rajpara. During an informal interaction with a group of women, all of them complained about the drinking and gambling habits of the male members in their families and how these addictions have become a marital issue for some families and have led to occasional violence.

#### The transformation of women’s work and opportunity

**Fig. 5:**
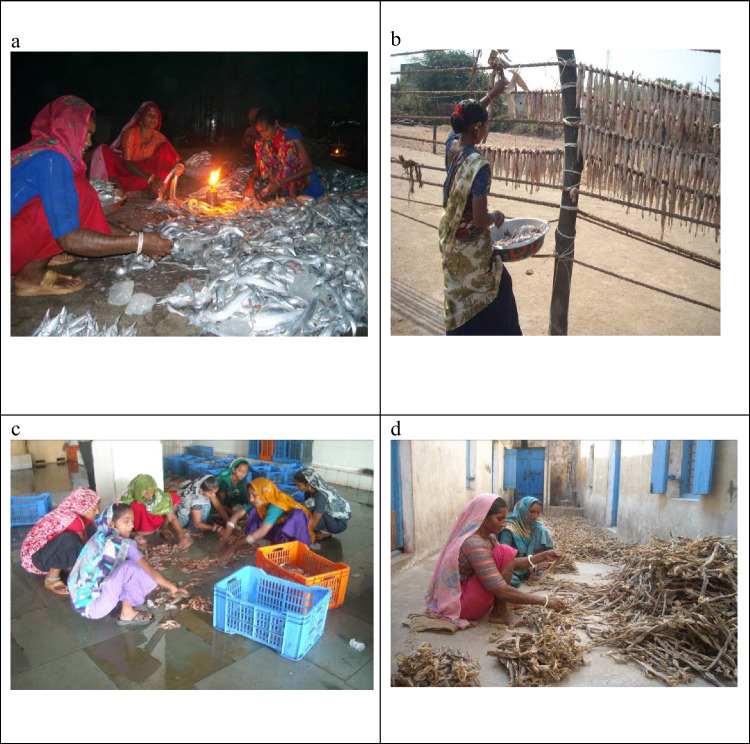
**a** Women sorting Bombay duck and ribbon fish at night under the open sky. **b** A Koli woman drying Bombay duck in the sun. **c** Young women sorting fish inside a processing unit. **d** Women sorting dried fish inside a dried fish processing unit. Photo credit: Rajib Biswal

Transitions in the bag net fishery have had contradictory impacts on the quality of work for women and men. In general, these changes have been positive for women, while, as noted above, men’s work has become increasingly onerous and stressful (Johnson et al. 2018). The expansion of the fresh fish industry and the relative decline of the dried fish trade, and the increase of production for commercial rather than subsistence purposes have reshaped women’s work in the fishery. Women spend relatively less time on traditional curing and sun-drying and more on sorting and cleaning to meet the labour demand from the fresh fish industry (Bhatta and Rao 2003). Women are generally employed by fish traders and boat owners on yearly contracts to engage in post-harvest processing work (Fig. 5c and d). The transition from a subsistence-based to a commercial fishery has provided financial autonomy to women compared to the earlier traditional dried-fish trade, which entailed intense yet unpaid work on the catch from household boats. Equally, women who are hired by the fish traders have access to better working conditions, such as protection from the sun and access to washrooms and drinking water. On the other hand, women who are hired by the boat owners or who work as part of the dried fish trade on a daily wage labour basis do not have access to these facilities and most of them have irregular working hours (Fig. 5a and b). Informant SR55 shared, ‘Whenever our boat arrives at the harbour, we have to be there. We do not see the time or whether it is day or night’. During the first author’s informal interactions, some women from the village complained about their hectic and erratic work schedule during the fishing season.

The social and economic dimensions of women’s lives have improved due to their increasing financial autonomy and heightened mobility. Informant SR62 observed, ‘The transformation in the fishery has changed our (women’s) lifestyle. Young women, employed in the fish processing units are making decent earnings. Women can afford more clothes and they have more options to choose from. We can afford to buy soaps and shampoos to clean ourselves, which feels good. Women are financially independent, and they are able to spend money for their own needs’. Many women have greater capacity to dress well and buy status symbols such as cell phones and gold jewelry. Women who work have better decision-making power within their families (Rubinoff 1999; Hapke 2001). For example, a few women have independently chosen their life partners and informed their parents about their marriage choices. This assertion of self-interest was very unusual even only 10 years before the research was conducted. Nonetheless, Saiyad Rajpara remains a male-dominated society, as is typical of South Asian fishing communities (Rubinoff 1999; Deb et al. 2015). The newly gained freedom and material status of women has triggered some social insecurity in Saiyad Rajpara. The most notable consequence of this has been a reported decrease in women’s marriageable age as parents seek to arrange their daughters’ marriages as early as possible to avert any kind of social embarrassment from potential elopement or unwanted pregnancies. The economic restructuring of the fishery has thus at once created new opportunities for women, but also led to a countervailing reaction to constrain that heightened freedom.

### Subjective wellbeing

During the market-driven boom phase, the fishing industry in Saiyad Rajpara flourished, and credit and employment opportunities for men and women expanded and were readily available. Men reflect on this period as one of great satisfaction as catches were good and provided a nearly year-round source of income. This was a period for them of increased income, when the standard of living for their families rose, and there was only moderate stress and risk. This was also a time of increased satisfaction for women, as they began to have access to their own income and their mobility increased.

For women, improved working conditions continue to translate into increased subjective wellbeing in the post-boom period. Many women, particularly young adolescent girls, are employed in various fish processing units within and outside the village. The increase in demand for more female labourers in various fish processing units has also resulted in increased mobility of women from many rural areas to the workplace (Reed et al. 2013). This ‘feminisation of migration’, as described by Weeratunge et al. (2010:9), has transformed the lives of women of the Koli fishing community in Saiyad Rajpara and neighbouring areas. The women’s group shared, ‘Currently, women are in much better condition than earlier times when we were young. Women at present can go here and there without much restriction which was not the case 30 to 40 years ago. Women have a voice in decision making, especially in marriage-related decisions, whereas that was not an option for the previous generation. The current generation is well informed, better dressed, and many ways smarter than the previous generation’. Many women feel empowered and emancipated by their increased income and mobility.

On the other hand, declining catches in recent years have made the life of fishermen more stressful. Many fishers complained about spending less time ashore and missing out important functions such as the birth of a child or the sudden death of a family member. Fishers noted the burden of long hours at the sea in an all-male environment as hindering normal life activities: physical and mental relaxation, sexual gratification, and the pursuit of personal interests. They also raised concerns about the riskiness of fishing in the unpredictable maritime environment. Nonetheless, perhaps speaking to the relational satisfactions of their profession, 46% of fishers said they were happy with their busy life, while 32% fishers had a neutral view towards their life, and 22% of fishers were unsatisfied. As noted above, alcohol consumption and use of tobacco products were common methods used by male fishers to cope with the stress of their occupations.

Transition in the bag net fishery in Saiyad Rajpara may have created a major opportunity for the local economy. Whether it has contributed to the subjective wellbeing of the fishers is, however, debatable. The material aspect of life has improved for most fishing households; however, fishing is subjectively allotted value that varies from individual to individual. Despite the enhancement of material and, in some cases, relational dimensions of wellbeing for male fishers, we judge that the transition in the fishery has reduced their overall quality of life due to the pressures of work and associated subjective frustrations (Biswal 2015).

## Conclusion

As we have tried to show in this paper, the social wellbeing approach offers a valuable way to tie change together with the lived experiences of men and women in small-scale fisheries. The relational quality of the social wellbeing approach is analytically powerful, particularly when linked to other social science approaches that share a similar epistemological commitment to the variabilities of experience and tensions inherent in social life. In this paper, we have pointed to political ecology as a complement to social wellbeing to ground our understanding of change, gender theory for deepening our understanding of how and why men’s and women’s experience of change have diverged, and interactive governance as a means to frame institutional constraints and political alternatives at present. In this last section of the paper, we summarise how a gender-theory informed approach to social wellbeing has helped us interpret women’s and men’s different recent experiences in the bag net fishery of Saiyad Rajpara before proposing some concluding reflections on implications for governance.

Saiyad Rajpara has mirrored at a slightly later date the dramatic market-oriented transition experienced by the fishers of Gujarat from the 1980s (Johnson 2001). The transition in Saiyad Rajpara has contributed to the material wellbeing of the residents of Saiyad Rajpara in areas such as food security, employment, income, and standard of living. The expansion of fishery has also benefitted people from neighbouring villages who are gainfully employed in this fishery and the many fish traders who link Saiyad Rajapara’s production to broader markets. The fishery’s growth has had negative impacts on the marine environment and mixed impacts on the subjective and relational wellbeing of Saiyad Rajpara’s inhabitants.

We have emphasised the gendered consequences of the transition in Saiyad Rajpara’s fishery through the changing interplay of material, subjective, and relational wellbeing. Here, consequences have bifurcated towards the positive for women and the negative for men. In this area, our research echoes Weeratunge et al.’s (2010) call to look at how globalisation and economic growth affect men and women differently. Women’s horizons, assets, and agency have all grown as an effect of the fisheries transition in Saiyad Rajpara, while men’s conditions of work and changes in men’s relationships have tended towards the less satisfactory, resulting in coping strategies for men that are sometime self-destructive.

Gujarat’s marine policy is growth-oriented and has traditionally emphasised commercial mechanised fishing (Armitage and Johnson 2006). Despite generating revenue and generating employment for many coastal communities, small-scale fisheries in Gujarat have low levels of collective organisation and suffer neglect by the state. Gujarat’s marine policy could do much more to accommodate and support small-scale fisheries. Consistent with the Small-scale Fisheries Guidelines (FAO 2015), there should be much more attention to the rights of small-scale fishers and to addressing the longstanding governance crisis of fisheries in Gujarat (Johnson 2001; Biswal et al. 2017). At the same time, it is important for small-scale fishers to become aware of their rights and to become more organised to, for example, advocate for themselves with the state and assert themselves against exploitation by fish traders. Our analysis of Saiyad Rajpara shows how state failure to engage effectively in fisheries governance has contributed to a key component of the decline of relational wellbeing for men in that fishing village. Our research also demonstrated the very important contribution of women to the fishery in Gujarat and suggests much greater state and civil society attention could be profitably oriented towards further supporting women’s economic and social roles. Our research also suggests that attention to the changing dynamics of relationships between women and men due to transitions in fisheries is merited to address emerging tensions as men’s and women’s quality of work and opportunities diverge.

The social wellbeing approach in conjunction with a political ecology informed analysis of change can help understand how the experience of men and women and other differently positioned actors in small-scale fisheries experience change. Research on fisheries in Gujarat needs to continue to document the wellbeing context and ongoing impacts of change. There remain pressing shortcomings in the way fisheries in Gujarat are understood, appreciated, and supported. Broader and deeper knowledge is needed about how male and female participants in the fishery collectively, and divergently, derive wellbeing from their engagement in fishing, fish processing, and fish trading, even as conditions change. Only if there is a shift to much better acknowledging and engaging with Gujarat’s small-scale fisheries, can those fisheries continue so successfully to provide economic, nutritional, and livelihoods benefits.

## Data Availability

N/A.

## Footnotes

1 Belton and Thilsted (2014) state that India ranks low in terms of the average per capita consumption of fish at only 5.4 kg/year due to cultural and religious reasons.

## Code availability

N/A.
